# Bilateral Renal Artery Thrombosis: A Case of Successful Kidney Recovery After Prolonged Anuria

**DOI:** 10.7759/cureus.30087

**Published:** 2022-10-09

**Authors:** Marta Filipa Lemos Mendes, Bárbara Ribeiro, Daniela Barros, Filipa Rodrigues, Sofia Marques

**Affiliations:** 1 Internal Medicine, Hospital de Braga, Braga, PRT; 2 Nephrology, Hospital de Braga, Braga, PRT; 3 Radiology, Hospital de Braga, Braga, PRT

**Keywords:** atrial fibrillation, hypertrophic cardiomyopathy, renal artery, thrombosis, anuria

## Abstract

Anuria suggests complete urinary tract obstruction, acute cortical necrosis, or massive vascular occlusion. We report a case of bilateral renal artery thrombosis in an 87-year-old woman admitted to the emergency department with abdominal pain, diarrhea, and anuria in the last 24 hours. Serum creatinine at admission was 5.87 mg/dl and urea was 100 mg/dl. Computed tomography showed renal artery thrombosis and partial splenic infarction. A conservative approach was performed with anticoagulation with warfarin. The patient recovered renal function and urine output months later.

## Introduction

Anuria may be suggestive of complete urinary tract obstruction, acute cortical necrosis, or massive vascular occlusion. Renal infarction is rare (prevalence estimated from autopsy studies at 14 per 1000 [1]) and underdiagnosed because the symptoms at the time of presentation are predominantly nonspecific. The two main causes of renal infarction are thromboembolism and thrombosis in situ; embolus usually originates from a thrombus in the heart or aorta and thrombosis in situ is usually due to an underlying condition of hypercoagulability or injury or dissection of a renal artery. Renal infarction is more common in patients with atrial fibrillation, cardiomyopathies, and/or valvular heart disease [2].

The authors report a case of renal infarction caused by bilateral renal artery embolism in a patient with paroxysmal atrial fibrillation and hypertrophic cardiomyopathy.

## Case presentation

**Figure 1:**
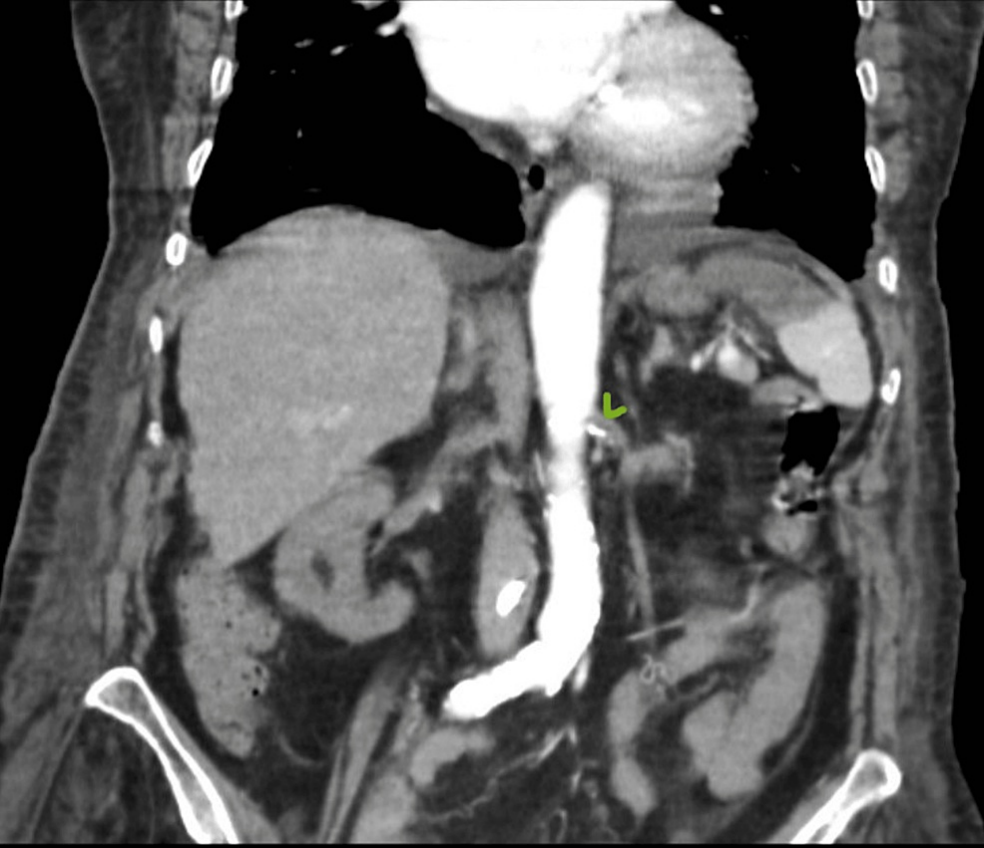
Ostium thrombosis of the left renal artery (coronal section)

**Figure 2:**
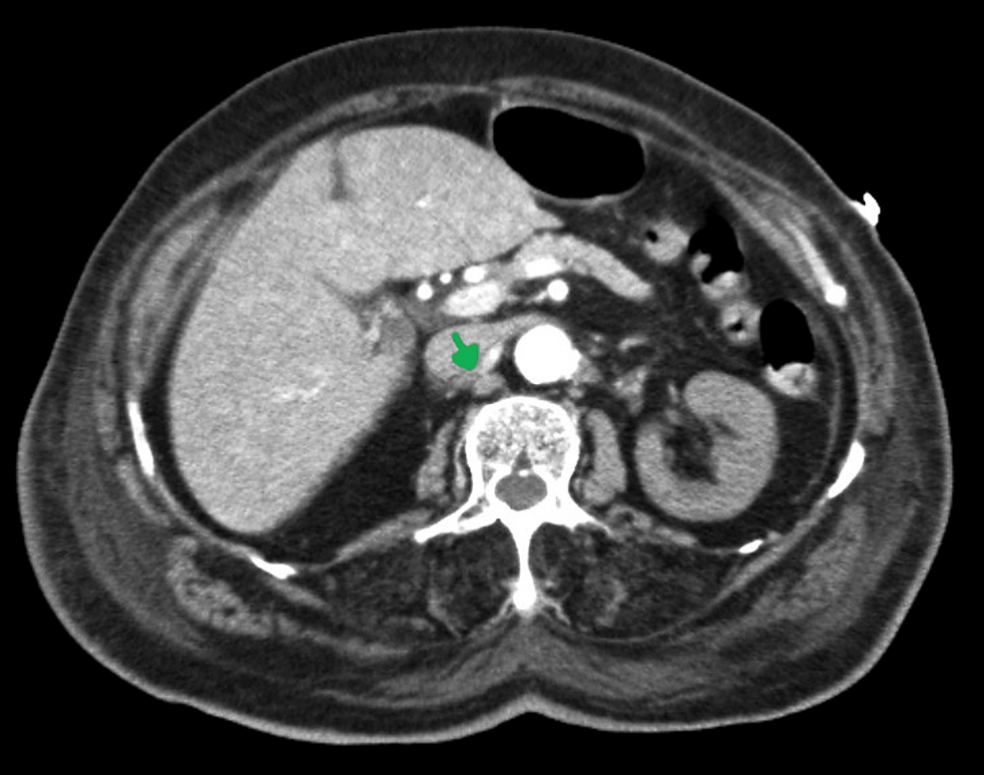
Thrombosis downstream of the right renal artery (sagittal section)

**Figure 3:**
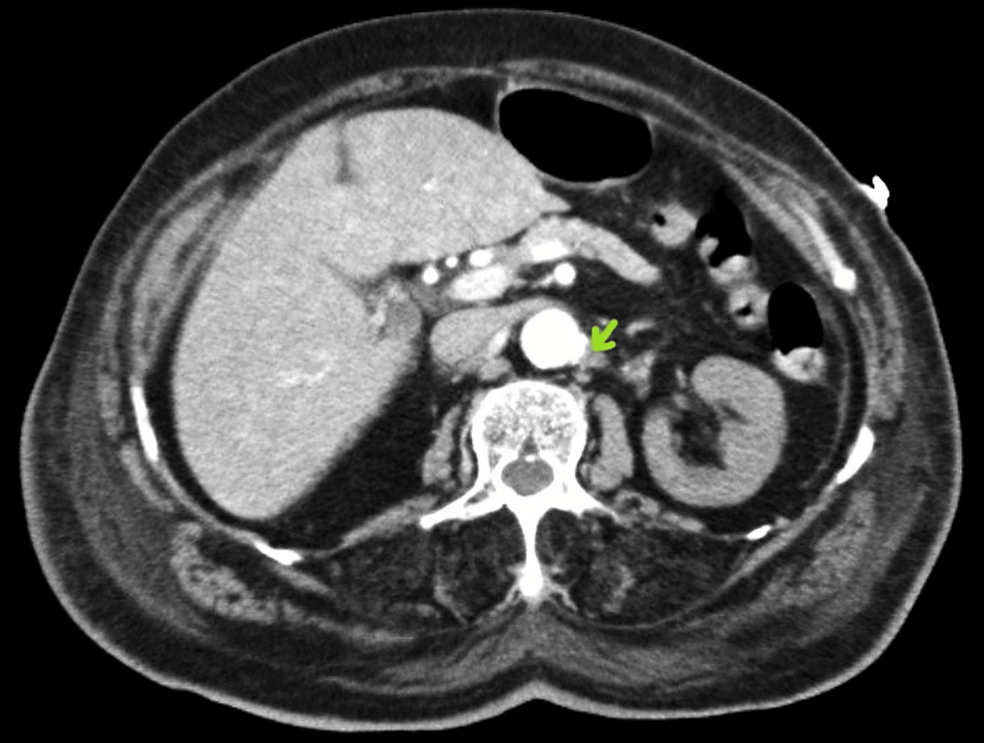
Ostium thrombosis of the left renal artery (sagittal section)

**Figure 4:**
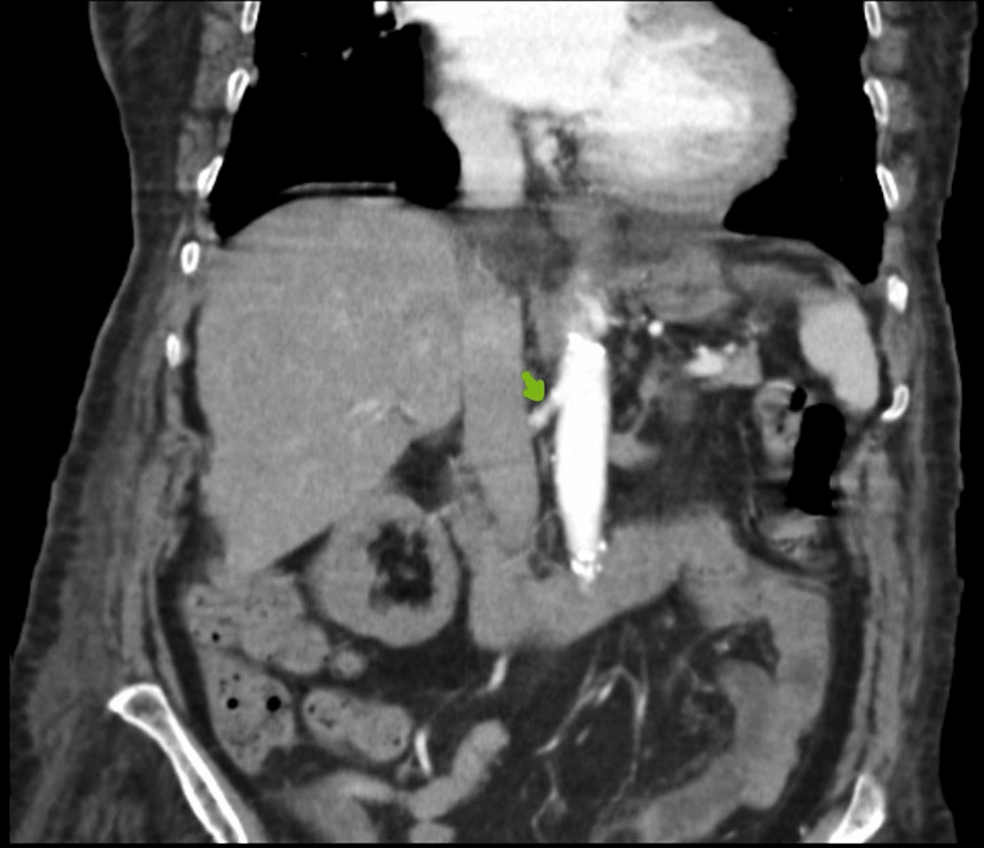
Proximal portion permeable right renal artery (coronal section)

We present a case of an 87-year-old woman with a history of hypertrophic cardiomyopathy, mild aortic stenosis, paroxysmal atrial fibrillation, and hypertension under perindopril, indapamide, lercanidipine, and nebivolol. She was admitted to the emergency department due to abdominal pain, diarrhea, and anuria for the past 24 hours. Six days earlier, she had been prescribed amoxicillin/clavulanic acid and ibuprofen for a respiratory infection and three days earlier, she attended medical care due to focal momentary neurological deficits presumably due to a transient ischemic attack (TIA). At the examination, her blood pressure was 170/80 mmHg and she complained of discomfort on abdominal palpation. Lab tests revealed an acute kidney injury (serum creatinine: 5.87 mg/dL; urea: 100 mg/dL) and elevated C-reactive protein (218 mg/L) and lactate dehydrogenase (LDH) (679 U/L). Urine could not be collected due to anuria. The kidney ultrasound revealed normal kidneys without hydronephrosis. The electrocardiogram showed sinus rhythm. Despite endovascular fluids, the patient remained in anuria, became hypervolemic, and required hemodialysis. To exclude vascular events, a CT scan was performed and showed a bilateral renal artery thrombosis and a partial splenic infarction (Figures 1-4). The echocardiogram (transthoracic and transesophageal) showed an inferior vena cava with 25 mm (without respiratory variability), predominant apical distribution of the hypertrophic cardiomyopathy, and this was considered a possible cause of the thromboembolism although no vegetation or thrombi was seen. Antiphospholipid syndrome was excluded (titers of anti-beta-2-glycoprotein, anticardiolipin antibodies, and lupus anticoagulant activity were negative at baseline and after three months). Endovascular treatment was not performed due to the uncertain time since infarction, and anticoagulation was started first with low-molecular-weight heparin and subsequently with warfarin (choice considered adequate because the patient was on dialysis). One month later, the patient developed diuresis, two months later, she no longer required dialysis, and at present time, she has a serum creatinine of 1.3 mg/dL.

## Discussion

Renal infarction should be strongly considered when presented with the following triad: persistent abdominal and/or flank pain, elevated serum LDH and/or hematuria, and risk of a thromboembolic event [3].

The occurrence of bilateral renal artery embolization with consequent acute renal failure is extremely rare, with most cases due to cardioembolism, particularly in patients with atrial fibrillation, cardiomyopathies, or valvular heart disease [3,4].

Given the rarity of this disease, there is insufficient evidence on therapeutic options. Thrombolytic therapy is typically reserved for patients diagnosed early in the disease, with the optimal port-to-treatment time being 90-180 minutes [5,6]. In the case presented, due to late diagnosis, endovascular treatment was not performed (>24 hours from the onset of symptoms), and anticoagulation was started. The cause of bilateral embolism of the renal arteries was considered to be a thromboembolism secondary to atrial fibrillation. The patient had a favorable clinical evolution with anticoagulation only and had an almost complete recovery of renal function.

## Conclusions

In this case of acute kidney injury, the previous diagnosis of paroxysmal atrial fibrillation, the presence of anuria, elevated serum LDH, previous history of TIA, and splenic infarction were important diagnostic clues of what was later confirmed by contrast-enhanced CT.

Despite prolonged anuria and apparent lack of viability of renal arterial circulation, which is why endovascular treatment was not performed, the patient had a very favorable clinical course with complete recovery of renal function.

Even so, the authors emphasize the need for early diagnosis to reduce the risk of progression to chronicity and improve the prognosis.
